# Frontal EEG Asymmetry of Mood: A Mini-Review

**DOI:** 10.3389/fnbeh.2017.00224

**Published:** 2017-11-06

**Authors:** Massimiliano Palmiero, Laura Piccardi

**Affiliations:** ^1^Neuropsychology Unit, IRCCS Fondazione Santa Lucia, Rome, Italy; ^2^Department of Biotechnological and Applied Clinical Sciences, University of L'Aquila, L'Aquila, Italy; ^3^Department of Life, Health and Environmental Sciences, University of L'Aquila, L'Aquila, Italy

**Keywords:** emotion, disposition, frontal asymmetry, mood induction, individual differences, depression, gender, pre-frontal cortex

## Abstract

The present mini-review was aimed at exploring the frontal EEG asymmetry of mood. With respect to emotion, interpreted as a discrete affective process, mood is more controllable, more nebulous, and more related to mind/cognition; in addition, causes are less well-defined than those eliciting emotion. Therefore, firstly, the rational for the distinction between emotion and mood was provided. Then, the main frontal EEG asymmetry models were presented, such as the motivational approach/withdrawal, valence/arousal, capability, and inhibition asymmetric models. Afterward, the frontal EEG asymmetry of mood was investigated following three research lines, that is considering studies involving different mood induction procedures, dispositional mood (positive and negative affect), and mood alterations in both healthy and clinical populations. In general, results were found to be contradictory, no model is unequivocally supported regardless the research line considered. Different methodological issues were raised, such as: the composition of samples used across studies, in particular, gender and age were found to be critical variables that should be better addressed in future studies; the importance of third variables that might mediate the relationship between frontal EEG asymmetries and mood, for example bodily states and hormonal responses; the role of cognition, namely the interplay between mood and executive functions. In light of these issues, future research directions were proposed. Amongst others, the need to explore the neural connectivity that underpins EEG asymmetries, and the need to include both positive and negative mood conditions in the experimental designs have been highlighted.

In these last decades, the cognitive neuroscience of emotion has enormously increased, aiming at improving the understanding of the biological basis of emotional processing in both healthy and clinical populations. A variety of approaches have been used so far, including functional Magnetic Resonance Imaging (fMRI). However, given the high temporal resolution of the electroencephalography (EEG), the change of EEG signals has been extensively used to detect real-time emotional processes that arise following a series of external/internal stimuli or events. One of the most prolific research lines has focused on the investigation of frontal EEG asymmetries of emotion and affect-related phenomena (e.g., mood). In this vein, moving from the rational that emotion and mood are distinct affective processes, the present mini-review was aimed at clarifying the EEG frontal asymmetry of mood. At the aim a selection of those EEG studies focused on mood induction, dispositional mood (e.g., positive and negative affect) and mood alterations in both healthy and clinical populations (e.g., depression and anxiety) were reviewed. Of course, the goal was not to systematically review all studies on the mood frontal asymmetry, but rather provide examples for the most important research lines in order to get insights about the current status of the research, in order to detect possible methodological- or theoretical-related issues and to draw possible future scenarios.

## Differences between emotion and mood

Emotion and mood are two distinct affective processes for different reasons. Beedie et al. (2005) revealed that eight themes were cited by both non-academics and academics (scientific literature). Excluding duration (emotion was evaluated both shorter and longer than mood) and function (intrinsic property to both processes), at least six reliable criteria were identified: causes, consequences, intentionality, intensity, physiology, and awareness of the cause. On the one hand, emotion involves specific causes, consequences on behavior, direction at something, high intensity, physical chemical response (e.g., adrenaline/fear), identification of the cause. On the other hand, mood is characterized by no specific causes, consequences on cognition, no specific direction at something, low intensity, psychological response and hormonal influences, no identification of the cause. In addition, emotion cannot be controlled (Ekman and Davidson, 1994), whereas mood can be controlled (Parkinson et al., 1996) and experimentally manipulated via different induction procedures, for example using music (e.g., Thompson et al., 2001; Palmiero et al., 2015, 2016). Emotion is mostly showed by facial expressions (Ekman, 1994), is clearly defined (Parkinson et al., 1996), whereas mood is hidden to others or expressed via body postures (Parkinson et al., 1996), and is more nebulous (Vallerand and Blanchard, 2000). Emotion is related to the heart and feeling, mood to the mind and thinking (Beedie et al., 2005). In addition, according to Scherer (2005) emotion is also characterized by response synchronization, that would play a key role on the preparation of the organism in order to face the emotional situation that has arisen by a specific cause; on the contrary, response synchronization is not important for mood because the organism must not prepare appropriate responses to unidentifiable eliciting causes.

## EEG frontal asymmetry of emotion: the basic models

The pioneristic frontal EEG asymmetry model (Davidson, 1983, 1993) supports the view that the activity of brain systems both moderates motivational trait tendencies to approach/withdraw novel emotional stimuli and mediate approach/withdrawal motivational tendencies underlying emotion. According to this model, an increase of the left prefrontal activity, either as a trait or as a state, is associated to approach-related emotions (e.g., positive), whereas an increase of the right prefrontal activity is associated to withdrawal-related emotions (e.g., negative).

According to the valence-arousal model (e.g., Heller, 1990, 1993; Berntson et al., 2011) the valence of emotions would be more important than the motivational tendencies: positive emotions are specifically associated with more left than right hemispheric activity, whereas negative emotions are associated with more right than left hemispheric activity.

In general, these two models diverge conceptually but overlap in terms of empirical predictions (Spielberg et al., 2008), that is, positive emotions are linked to approach-related motivation, whereas negative emotions to withdrawal-related motivation. With a few exceptions (e.g., Mller et al., 1999; Elgavish et al., 2003), the most of studies confirmed these asymmetry models (for review see Davidson et al., 2000; Coan and Allen, 2004). However, results collected with anger, which involves a negative valence but also an approach tendency (e.g., Berkowitz, 1999), raised doubt on the assumptions of the asymmetry models. Indeed, different studies demonstrated that anger yielded an increase of left rather than of right frontal EEG activity (e.g., Harmon-Jones, 2004a; Hewig et al., 2004; Gable and Poole, 2014; for a review see Harmon-Jones, 2004b). Collectively, these results show that EEG frontal asymmetry reflects the direction of the motivation rather than the valence of emotion.

More recently, Coan et al. (2006) proposed the capability model, which basically posits that, besides affective dispositions under resting condition, the situational variable plays a key role on the frontal EEG asymmetry. In other words, frontal EEG activity would rely on specific emotional contexts and individuals' capacity to respond emotionally (approaching vs. withdrawal responses) or to inhibit responses to the situation that has contributed to elicit emotions.

Yet, moving from the evidence that inhibitory processes are very important for emotional asymmetries (Jackson et al., 2003; Davidson, 2004; Coan et al., 2006), Grimshaw and Carmel (2014) proposed the asymmetric inhibition model, by which asymmetries can be interpreted in terms of executive control: mechanisms in left frontal cortex would inhibit negative distractors, whereas mechanisms in right frontal cortex would inhibit positive distractors. Different studies supported these predictions. For example, difficulty in releasing attention from negative stimuli was found to rely on low left frontal activity, as occurs in depression and anxious arousal (e.g., Cisler and Koster, 2010), whereas difficulty in inhibiting positive distractions was found to rely on low right frontal activity, as occurs in poor self-regulation and addiction (e.g., Goldstein and Volkow, 2011).

## Frontal EEG asymmetry of mood

Three research lines were followed, that is studies exploring the relationships between frontal EEG asymmetries and: (1) mood states induced by different experimental procedures (e.g., film clips, music, faces); (2) dispositional mood (positive and negative affect); (3) mood alterations in both healthy and clinical populations (see Table 1 for details).

**Table 1:** List of studies for each research line.

**MOOD INDUCTION**			
**Study**	**Method**	**Subjects**	**Main result**
Tucker et al., 1981	Textbook descriptions of euphoria and depression	10 (6 females); Students	Depression: ↑RFA
Tomarken et al., 1990	Positive and negative film clips Subjective emotional responses to film clips	32 females 17–41 years	NA: ↑RFA
Wheeler et al., 1993	As in Tomarken et al. (1990), but baseline EEG recorded twice 3 weeks apart; subjects with stable patterns of asymmetry	26 females 17–21 years	NA: ↑RFA; PA: ↑LFA
Gotlib et al., 1998: Study 2	Sad mood induced using negative music Non-verbal fluency task for control condition	59 females divided in: high vulnerable ↓LFA; low vulnerable ↑LFA	No relationship between EEG asymmetry, mood, cognitive functioning
Gale et al., 2001	Pictures of sad and happy faces Eysenck Personality Inventory Subjective emotional response to faces	30 females 18–36 years	Negative mood: ↑LFA Extraversion: ↑RFA for PA; Neuroticism: ↑left/right ratios and ↓RFA
Dennis and Solomon, 2010	Emotion regulation: self-reported change in negative mood induced using fearful, sad, neutral film clips; attention interference in a task with mood congruent emotional distractors	66 (40 females) 18–59 years	↑FA during mood inductions vs. baseline: more emotion regulation No significant asymmetry
Kop et al., 2011	Recall of happy and anger incidents	20/30 (55% females) Mean age 25 years	Positive mood: RFA
Rodriguez et al., 2015	Sadness induced while participants virtually navigated through a park by music, Velten self-statements, pictures, movies	24 (12 females) 19–36 years 9 controls; 9 reappraisal; 9 expressive/suppression	Sadness: ↑RFA only in controls
Warden-Smith et al., 2017	Light-pleasant smell to optimize positive psychophysiological benefit	24 for stage 1 64 for stage 2 NFA (difference between Alpha-wave activity in the right and left frontal hemispheres) and PFA groups.	Negative group (NFA): ↓RFA and ↑LFA No significant effect on the positive group
**DISPOSITIONAL MOOD**			
**Study**	**Mood Measures**	**Subjects**	**Main Results**
Tomarken et al., 1992a	Baseline EEG on two occasions 3 weeks apart; PANAS	90 females 17–21 years	LFA: ↑PA, ↓NA compared with RFA
Tomarken et al., 1992b	As in Tomarken et al. (1992a)	85 females 17–21 years	As in Tomarken et al. (1992a)
Jacobs and Snyder, 1996	PANAS; BDI	40 males 18–53 years	↑LFA: ↓NA and ↓BDI
Sutton and Davidson, 1997	Baseline EEG on two occasions 6 weeks apart PANAS first session; BIS/BAS scales second session	46 (23 females) 18–22 years	No relationship between Pre-Frontal EEG asymmetry and PA or NA
Hagemann et al., 1999	Transient Mood assessed on a 0-9 scale; PANAS Eysenck Personality Questionnaire	36 (24 females) Mean age 24.7	Subjects with ↑NA: ↑LTA (but not LFA) than in subjects with ↓NA. No relation between asymmetry and PA
Hall and Petruzzello, 1999	PASE; STAI-Y2; PANAS; GDS; SWLS	41 (26 females) Mean age 68.7	LFA predicted PA High-active group: FA predicted affective valence and SWL Low active group: FA predicted NA
Mikolajczak et al., 2010	Trait Emotional Intelligence Questionnaire	31 (25 females) Mean age 22.4	No relationship between EEG FA and well-being subscale
**MOOD ALTERATIONS**			
**Study**	**Method**	**Subjects**	**Main Results**
Schaffer et al., 1983	BDI	15 (10 females)	↑RFA: ↑BDI
Henriques and Davidson, 1990	BDI; Hamilton Rating Scale for Depression	14 (6 previously depressed) Mean age previously depressed 37.4 Mean age controls 34.7	↓LFA in previously depressed subjects relative to controls; no difference between groups on self-reported emotional state
Henriques and Davidson, 1991	BDI; Hamilton Rating Scale for Depression	28 (18 females) 15 currently depressed: 33–57 years 13 controls: 40–61 years	↓LFA in currently depressed subjects relative to controls; no correlation between FA and state ratings of emotion at the time of the baseline recording and depression
Allen et al., 1993	Pre-post bright light treatment	8 females (4 with Seasonal Affective Disorder)	↓LFA in Seasonal Affective Disorder relative to Control
Tomarken and Davidson, 1994	MC; STAI; BDI	90 females	Repressors ↑LFA than non-repressors No asymmetry difference between high-anxiety and low-anxiety, high-depression and low-depression groups
Gotlib et al., 1998: Study 1	Inventory to Diagnose Depression (IDD); Lifetime version of the IDD; 2 modules of the DSMIII-R: Major Depressive Disorder and Dysthymic Disorder	77 females 30 never depressed; 31 previously depressed; 16 currently depressed	↓LFA in currently depressed and previously depressed subjects compared to never depressed subjects
Reid et al., 1998	Study 1: BDI Study 2: DSM-III-R	Study 1: 36 females (17 depressed) Mean age 18.53 Study 2: 27 females (13 depressed) Mean age 27.54	No frontal asymmetry between depressed and non-depressed subjects in both studies
Papousek and Schulter, 2002	Study 1: Anxious tension anchored 17-point bipolar rating scale; Negative mood assessed by an adjective checklist Study 2: separate scales for state depression and state anxiety	Study 1: 56 (30 female): 18–36 years Study 2: 128 (68 female): 18–31 years	Anxiety, tension, and depression decrease when frontopolar activation asymmetry shifted to the right hemisphere
Mathersul et al., 2008	Depression Anxiety Stress Scales (DASS-21)	428 (214 females) 18–60 years	↑RFA associated to anxious arousal ↑LFA associated to anxious apprehension and to non-depression Symmetrical frontal activity associated to depression and comorbidity

*↑, Increased; ↓, Decreased; LFA, Left Frontal Activation; RFA, Right Frontal Activation; LTA, Left Temporal Activation; NFA, Negative Frontal Asymmetry; PFA, Positive Frontal Asymmetry; PANAS, Positive and Negative Affect Schedule; NA, Negative Affect; PA, Positive Affect; EEG, Electroencephalography; BIS, Behavioral Inhibition System; BAS, Behavioral Activation system; PASE, Physical Activity Scale for Elderly; STAY-Y2, State-Trait Anxiety Inventory (Trait); GDS, Geriatric Depression Scale; MC, Marlowe-Crowne Social Desirability Scale; SWLS, Satisfaction with Life Scale*.

## EEG frontal asymmetry and induction of mood state

In one of the first studies, Tucker et al. (1981) revealed that the induced euphoria mood state generated symmetry, whereas the induced depression mood state was associated with greater activation of the right frontal lobe. Tomarken et al. (1990) also found that subjects' asymmetry predicted the level of negative affect in response to the negative film clips, which was related to greater activation in the right hemisphere. Using data from those subjects with stable patterns of asymmetry across 3-weeks period, Wheeler et al. (1993) replicated Tomarken et al. (1990) results, and also found greater left frontal activation associated with reports of more intense positive affect in response to the positive films. Rodriguez et al. (2015) also found significant activations in different right frontal regions due to the induction of negative mood in the control group but not in cognitive reappraisal and expressive suppression groups. Collectively, these results suggest that hypoactivation of the left frontal region is an individual predisposition that underlies elevated responsivity to negative stimuli, increasing the risk for mood disorders, especially depression. However, Gale et al. (2001) revealed greater activation of the left frontal hemisphere with negative mood, whereas participants' personality (and gender of the face viewed) mediated the direction of the differentiation between positive and negative mood in the right hemisphere. Indeed, extraverts showed greater right hemisphere activation for positive affect, whereas, neurotics showed increased left/right ratios and less activated right hemisphere. Kop et al. (2011) also found increased right frontal activation during induced positive mood induction, which was associated with a decrease in low frequency/high frequency ratio of the heart rate variability. Interestingly, Warden-Smith et al. (2017) showed that a positive mood induction yielded a decrease of right frontal asymmetry and an increase of left frontal asymmetry in negative alpha fontal group, as if a change in alphawave activity in the direction of positive affect occurred in people susceptible to negative affect. Yet, Gotlib et al. (1998) found in the study 2 that frontal EEG asymmetry was unrelated to mood reactivity and cognitive functioning. Dennis and Solomon (2010) also found that induced fear and anger were not related to greater right frontal asymmetry, but rather to bilateral activity.

## EEG frontal asymmetry and dispositional mood

In one large research project (e.g., Tomarken et al., 1992a,b), females with stable greater right frontal activation across two different sessions reported increased Negative Affect (NA), whereas females with stable left frontal activation reported increased Positive Affect (PA). However, Jacobs and Snyder (1996) only revealed that left lateral-frontal activation yielded lower score of NA in men, whereas Hall and Petruzzello (1999) showed that left frontal activation predicted PA in older adults of both sexes. In addition, other studies failed to observe significant relationships between the affective dimensions and frontal asymmetry in a sample of both sexes (e.g., Sutton and Davidson, 1997; Hagemann et al., 1999). More recently, also Mikolajczak et al. (2010) found that frontal EEG asymmetries were not related to the factor of wellbeing, which is a trait pertaining to dispositional mood. In addition, in the attempt to support more specifically the assumption of an asymmetry/personality relationship, Hagemann et al. (1999) found that while extraversion correlated with positive affect scores, neither extraversion nor neuroticism correlated with any of the EEG measures.

## EEG frontal asymmetry and mood alterations

Comparing high vs. low scorers on the Beck Depression Inventory (BDI) on measures of resting EEG activation asymmetry, Schaffer et al. (1983) revealed that depressed subjects yielded greater right frontal activation than non-depressed subjects. In this direction, less left frontal activation was found in a sample of six euthymic individuals with a past history of depressive episodes relative to healthy subjects (Henriques and Davidson, 1990), in currently depressed (Henriques and Davidson, 1991; Gotlib et al., 1998) and previously depressed subjects (Gotlib et al., 1998), as well as in dysphoric patients with bipolar seasonal affective disorder relative to non-depressed controls, both before and after successful phototherapy (Allen et al., 1993). These results support the view that hypoactivation of the left frontal region represents a marker for mood disorders. However, once again contradictory results have been collected across years. For example, subjects classified as repressors showed relative left anterior cortical activation than non-repressors (Tomarken and Davidson, 1994), no asymmetry differences were not found between high-depression and low-depression groups using both Beck Depression Inventory scores (Tomarken and Davidson, 1994; Reid et al., 1998) and subjects diagnosed with DSM-III-R depression relative to controls (Reid et al., 1998). In addition, no difference was found between high-anxiety and low-anxiety groups (Tomarken and Davidson, 1994). Interestingly, negative spontaneous mood (e.g., anxiety, tension, depression) was found to decrease across two different sessions when frontopolar activation asymmetry spontaneously shifted to the right hemisphere (Papousek and Schulter, 2002). More recently, Mathersul et al. (2008) found that anxious arousal subjects showed higher right frontal asymmetry, anxious apprehension and non-depression subjects showed higher left frontal asymmetry, whereas symmetry was found for depression and comorbid subjects.

## Conclusions

From the studies reviewed on the EEG correlates of mood it appears that, regardless the research line considered, there are contrasting results that cannot be unequivocally interpreted according to one frontal asymmetry model rather than to another. The motivational approach/withdrawal and valence/arousal models appear to be the most supported ones (Tucker et al., 1981; Schaffer et al., 1983; Henriques and Davidson, 1990, 1991; Tomarken et al., 1990, 1992a,b; Allen et al., 1993; Wheeler et al., 1993; Gotlib et al., 1998—Study 1; Mathersul et al., 2008; Rodriguez et al., 2015; Warden-Smith et al., 2017). However, it is difficult to disentangle the contributions of specific studies to the two models given that the models overlap in terms of empirical predictions (Spielberg et al., 2008). The most of these studies might be also explained in light of the inhibition model of asymmetric differences, given that they revealed right frontal asymmetry or hypoactivation of the left hemisphere for negative mood, as if positive or approach-related distractors would be inhibited when there is a predisposition that supports elevated responsivity to negative stimuli. In addition, the capability model might also explain the most of results (e.g., Dennis and Solomon, 2010), as individual dynamic differences that are challenged by arousing situations, such as those relying on mood induction procedures. Nevertheless, the extent to which this model is appropriate to explain results when the situational variable is absent (e.g., dispositional mood) is unclear. Finally, some studies found results that do not fit with the models discussed (e.g., Papousek and Schulter, 2002; Kop et al., 2011), whereas other studies found frontal EEG asymmetry unrelated to mood (Tomarken and Davidson, 1994; Sutton and Davidson, 1997; Gotlib et al., 1998—Study 2; Reid et al., 1998; Hagemann et al., 1999; Mikolajczak et al., 2010).

These contradictory results depend on different reasons. Following Hagemann et al. (1998), firstly results vary according to methodological variables, such as different measurement procedures of asymmetry and affective variables. Secondly, it also appears that sample should be better composed. Indeed, different studies reviewed used only females (e.g., Tomarken et al., 1990; Wheeler et al., 1993; Gotlib et al., 1998; Reid et al., 1998; Gale et al., 2001), or much more females than males (e.g., Dennis and Solomon, 2010; Mikolajczak et al., 2010); one study enrolled only males (Jacobs and Snyder, 1996), and one study reported no information about gender (Warden-Smith et al., 2017). Only recently studies have increased the interest in gender-related brain mechanisms and cerebral lateralization subserving emotional processing (e.g., Gasbarri et al., 2006, 2007; Arnone et al., 2011). In particular, unpleasant stimuli (negatively valenced IAPS pictures) were found to elicit higher P300 amplitude and shorter P300 latency at left frontal site than pleasant and neutral stimuli in women than in men, while a stronger P300 component was elicited in the right hemisphere in men compared to women (e.g., Gasbarri et al., 2007; Arnone et al., 2011). In addition, participants' age might also be another confounding factor because different wide age ranges are reported across studies, even including over 50 (e.g., Jacobs and Snyder, 1996; Dennis and Solomon, 2010) or 60-year people (e.g., Hall and Petruzzello, 1999; Mathersul et al., 2008).

Thirdly, the relationships between frontal asymmetries and mood are also mediated by third variables that have been rarely considered beyond personality (e.g., Gotlib et al., 1998), emotion regulation-capabilities (e.g., Dennis and Solomon, 2010). For example, Hall and Petruzzello (1999) found that in older adults the relationships between frontal brain activity and dispositional affect is influenced by physical activity. This leads to suppose that although mood is generally associated to mind and thoughts, bodily states might also play a key role. Indeed, mood (and of course emotion—e.g., Neal and Chartrand, 2011; Palmiero and Borsellino, 2014) has been described as an embodied experience (e.g., Veenstra et al., 2016). At our knowledge, only Kop et al. (2011) included the measure of the heart rate variability in the study of EEG correlates of mood.

Therefore, the interplay between cognition and emotion should also be considered when studying the EEG asymmetries of mood. Cognition and emotion interact in prefrontal cortex. In particular, according to Grimshaw and Carmel (2014), the left dorsolateral prefrontal cortex (dlPFC) should inhibit negative distractors, whereas the right dlPFC should inhibit positive distractors. Consistent with this prediction, Compton et al. (2003) revealed the presentation of negative words in an emotional Stroop task yielded increased activation in the left dlPFC. Yet, different studies revealed that failures to recruit the left dlPFC during negative distractions are due to mood alterations, which yield higher activation of the right dlPFC (e.g., Engels et al., 2010). In this vein, it appears that frontal EEG asymmetries of mood must be also considering the underlying neural network organization.

In light of these issues, inferences drawn from data previously discussed are potentially limited by the scarce research examining EEG correlates of mood using standard procedures and samples, as well as the interplay with third variables and cognition. Then, frontal EEG asymmetries of mood might be better understood considering the extent to which parietal, temporal, and occipital asymmetries are also investigated. Indeed, Hagemann et al. (1999) showed significant greater relative left activation in the temporal lobe (but not in frontal lobe) in participants of both sexes with high negative affect than in participants with low negative affect. This means that also the neural connectivity between different brain areas should be investigated using more sophisticated neuroimaging approaches. Yet, given that the majority of studies used only negative stimuli, it is important that future research includes in the paradigm both positive and negative mood conditions, unless it is impossible to determine the extent to which hemispheric differences are related to valence.

In conclusion, pursuing more systematically the investigation of EEG asymmetries of mood adopting a wider perspective seems to be mandatory in order to achieve more consistent and reliable outcomes.

## Author contributions

MP collected studies and write up the minireview. LP contributed to write up the mini-review.

### Conflict of interest statement

The authors declare that the research was conducted in the absence of any commercial or financial relationships that could be construed as a potential conflict of interest.
